# The cross-talk between matrix metalloproteinase-9, RANKL/OPG system and cardiovascular risk factors in ovariectomized rat model of postmenopausal osteoporosis

**DOI:** 10.1371/journal.pone.0258254

**Published:** 2021-10-05

**Authors:** Maha Sabry, Seham Mostafa, Samaa Kamar, Laila Rashed, Suzanne Estaphan

**Affiliations:** 1 Faculty of Medicine, Physiology Department, Cairo University, Giza, Egypt; 2 Faculty of Medicine, Histology and Cell Biology Department, Cairo University, Giza, Egypt; 3 Histology Department, Armed Forces College of Medicine, Cairo, Egypt; 4 Faculty of Medicine, Biochemistry Department, Cairo University, Giza, Egypt; 5 ANU Medical School, Australian National University, Canberra, Australian Capital Territory, Australia; Karpagam Academy of Higher Education, INDIA

## Abstract

Epidemiology and pathogenesis of cardiovascular diseases (CVD) and osteoporosis are strikingly overlapping. This study presents matrix metalloproteinase-9 (MMP-9), as a simple molecular link more consistently associated with the pathophysiology of both osteoporosis and CVD risk factors. 40 adult female rats were randomly distributed into 4 groups [control sham-operated, untreated osteoporosis, carvedilol-treated osteoporosis and alendronate-treated osteoporosis]. After 8 weeks, blood samples were collected to estimate Lipid profile (Total cholesterol, HDL, Triglycerides), inflammatory markers (IL-6, TNF alpha, CRP and NO), and Bone turnover markers (BTM) (Alkaline phosphatase, osteocalcin and pyridinoline). The tibias were dissected to estimate MMP-9 and NF-kB gene expression, OPG, RANKL levels and for histological examination. Induction of osteoporosis resulted in a significant elevation in BTM, inflammatory markers and dyslipidemia. MMP-9 was significantly elevated and positively correlated with BTM, inflammation and dyslipidemia markers. Carvedilol and alendronate exerted a bone preservative role and attenuated dyslipidaemia and inflammation in accordance with their respective effect on MMP-9.

## Introduction

Menopause has an average onset around 50s, with increasing longevity. It could be considered a midlife event that presents an enormous healthcare problem due to its long-term complications. Osteoporosis (OP) affects 1 in 3 women, hip fracture has a high morbidity and mortality, and, cardiovascular disease (CVD) is the first cause of women death worldwide [1].

Osteoporotic patients have been found to be more prone to attain ischaemic heart diseases and likewise patients with CVS have been shown to be more prone for osteoporosis. Therefore, researchers suggested a causal relationship between both pathologies [2–4].

There is a tremendous need to identify the molecular link between both conditions in order to reach a treatment capable to efficiently address them simultaneously.

In fact, the association between osteoporosis and cardiovascular disease found in most epidemiological and pathophysiological studies necessitates a novel approach for evaluating osteoporosis and cardiovascular pharmacological treatment based on their potential benefits to moderate their counterpart disease respectively.

Of the components that perhaps best illustrate the bone-arterial wall biological linkages, osteoprotegerin (OPG) [5] has attracted the most attention. Yet, considerable controversy still exists regarding the role of OPG/RANKL/RANK/TRAIL in cardiovascular disease. RANKL has been shown to increase total matrix metalloproteinase activity in human fibroblasts, which indicates a matrix-degrading net effect [6]. Under high RANKL/OPG ratios, OPG enhances the RANKL-mediated effects on Matrix metalloproteinase (MMP) levels in vascular SMC, and the opposite happens under low RANKL/OPG ratios. Intriguingly, OPG has been shown to exert chemo tactic properties, and smooth muscle cells incubated with OPG showed increased apoptosis, interleukin 6, MMP-2 and MMP-9 levels and impaired cell proliferation [7]. There is as yet no hypothesis unifying the apparent dichotomy in the nature of OPG/RANKL/TRAIL noted in animal and human studies [8].

MMPs represent a crucial downstream molecule that mediate the destructive action of OPG/RANKL/TRAIL system. MMPs are proteases that participate in the organized degradation of the extracellular matrix (ECM) and hence play essential physiological roles, such as cardiovascular and bone tissue remodelling [9].

Considering this relationship between osteometabolic and cardiovascular pathologies and MMPs, altogether with the complexity of OPG/RANKL/TRAIL, MMP-9 could represent the novel molecular link needed as a marker and most importantly as a target for an efficient pharmacological treatment to both conditions simultaneously.

Our group has showed Matrix Metalloproteinase 9 (MMP-9), as a proposed link between atherosclerosis and osteoporosis in atherosclerotic rat model [10]. How consistent these results could be in postmenopausal osteoporosis rat model attired our interest.

### Aim of the work

The present work thus aimed to:

- Confirm the role of MMP9 in the pathogenesis of osteoporosis through examining the effect of induction of osteoporosis on MMP-9 gene expression in tibias and its correlation to OPG/RANKL axis components and bone turnover markers.
- Explore MMP9 as a novel link tying up osteoporosis with cardiovascular risk through investigating the correlation between MMP-9 and dyslipidaemia, inflammatory markers in addition to bone turnover markers in the osteoporotic rats.
- Evaluate the possible protective effect of carvedilol, a third generation B-blocker used for treatment of coronary atherosclerosis, on markers of osteoporosis and exploring its effect on MMP-9 (being our proposed key linking molecule) and comparing its probable effect against bisphosphonate (a known anti-osteoporosis drug).

## Materials and methods

### Experimental animals & groups

This study was carried out in the animal house of National Cancer institute, Cairo University. 40 adult female albino rats, 12 weeks of age, weights ranging from 150–200 gram were included in the study.

Rats were purchased and placed under ordinary living conditions in the animal house (temperature of 25±1ºC; 12-hour light/dark cycle). They were housed in wire mesh cages in groups of 4 at their arrival and allowed to accommodate to their new environment for 1 week. All rats had free access to water and food^1^. All procedures performed in studies involving animals were in accordance with the ethical standards and the recommendations for the proper care and use of laboratory animals and approved by the Institutional Ethical Committee of Cairo University (CU/III/F/61/17).

Animals were randomly divided into the following groups:

#### Group 1 (control): Sham ovariectomy group (n = 10)

Rats in this group underwent the same surgical steps of ovariectomy without surgical removal of the ovaries, the ovaries were lifted up and returned to their original position [11]. They were housed in standard cages (28 x 40) with the same cagemates and fed on standard laboratory rat chow^1^ for the whole 8 weeks duration of the study.

Osteoporosis was induced in the remaining rats by ovariectomy via a ventral abdominal transverse incision while the rats were anesthetized using ketamine (80mg/kg) and xylazine (10mg/kg), intraperitoneally [12].

The ovariectomized rats were further subdivided into 3 groups each comprising 10 rats.

#### Group 2 (untreated osteoporotic)

Rats received daily vehicle of 0.5 ml saline by oral administration for 8 weeks.

#### Group 3 (Carv-treated)

Rats were treated with carvedilol^2^ (10mg/kg) daily by oral gavage for 8 weeks [13].

#### Group 4 (Alendronate-treated)

Rats were treated with alendronate sodium^3^ (3mg/kg) daily by oral gavage for 8 weeks [14].

^1^ Composition of standard rat chow: 5.4% fat, 53.8% carbohydrate, 21.9% protein, 2.9% fibre mixture of minerals and vitamins obtained from the animal house [15].

^2^Carvedilol (Coreg) drug was provided in the form of tablets from Sandoz Company, Tablets were dissolved in saline and administrated orally to rats according to its weight, at a dose of (10mg/kg/d) [13].

^3^Fosamax (sodium alendronate): drug was provided in the form of tablets from Merck sharp & Dohme (MSD) Company, USA. Tablets were dissolved in saline and administrated orally to rats according to its weight, at a dose of (3mg/kg/d) [14].

### Experimental measurements

At the end of the 8 weeks experimental period, and after an overnight fast, blood samples were collected from retro-orbital plexus for estimation of Lipid profile (Total cholesterol, HDL, TGs), Inflammatory markers (IL-6, TNF alpha, CRP and NO) and Bone turnover markers (Alkaline phosphatase and osteocalcin, bone formation markers, and pyridinoline, bone resorption marker).

Rats were then euthanized by decapitation, and the tibias were dissected carefully. The right Tibia samples were frozen in liquid nitrogen and stored at -80C for Estimation of Metalloproteinase 9 gene expression, NF KB gene expression by PCR and OPG, RANKL protein expression by ELISA test. While the left Tibia samples were fixed in 4% neutral buffered formaldehyde. Decalcification was performed in ethylene diaminetetra-acetic acid (EDTA) solution. The decalcified specimens were then processed for paraffin blocks and serial transverse sections from the diaphysis were obtained for H&E and Masson’s trichrome stain examination.

### Concise methodology

#### Biochemical measurements

Serum cholesterol [16] and plasma Triglycerides were measured by quantative–Enzymatic–Colorimetric method. [17] HDL- cholesterol is obtained through selective precipitation of LDL and VLDL lipoproteins, thus HDL lipoproteins remain in solution [18].

Serum IL-6 [19], TNF- alpha [20], alkaline phosphatase (ALP) [21], RANKL [22] and OPG [23] protein expression were estimated through sandwich ELISA technique. Serum levels of Osteocalcin (OC) [24] and pyridinoline [25] were estimated through the competitive enzyme immunoassay technique. Nitric oxide was determined in serum according to the method of Miranda et al. [26].

MMP-9 and NF-KB gene expression were detected by real time Quantitative polymerase chain reaction (real time-PCR) in rats tibias [27].

*Quantitative real time PCR*. RNA extraction: Total RNA was isolated using Qiagen tissue extraction kit (Qiagen, USA) according to instructions of manufacture.

cDNA synthesis: The total RNA (0.5–2μg) was used for cDNA conversion using high capacity cDNA reverse transcription kit (Fermentas, USA).

Real-time qPCR using SYBR Green I: Real-time qPCR amplification and analysis were performed using an Applied Biosystem with software version 3.1 (StepOne^™^, USA). The qPCR assay with the primer sets were optimized at the annealing temperature. The primer sequence was shown in Table 1.

**Table 1:** The primer sequence of the studied genes.

	Primer sequence
**MMP-9**	Forward primer: 5-GTGGGAGAAAGTTTGCCAGG-3 Reverse primer:5- GTAGGAAGAGAGGGAAGAGG-3
**NF.KB**	Forward:5’- GGT TCC CTG GCA TAA TCT GA -3 Reverse:5’- GTC ATC GAG ACC CCA AGG TA -3
**Beta actin**	Forward 5′-ATCACCATCTTCCAGGAGCG -3′ Reverse 5′-CCTGCTTCACCACCTTCTTG-3′

Calculation of Relative Quantification (RQ) (relative expression): The relative quantitation was calculated according to Applied Bio system soft using the following equation according to Livak & Schmittgen [28], RQ = **2**^**−ΔΔCt**^.

Where, ΔΔ Ct = ΔCt (test samples)– ΔCt (calibrator samples)

$$\Delta \text{Ct}\;\left(\text{test}\;\text{samples}\right)\;=\;\text{Ct}\;\left(\text{target}\;\text{gene}\;\text{in}\;\text{tests}\right)\;–\;\text{Ct}\;\left(\text{reference}\;\text{gene}\;\text{in}\;\text{tests}\right)$$

$$\Delta \text{Ct}\;\left(\text{calibrator}\;\text{samples}\right)\;=\;\text{Ct}\;\left(\text{target}\;\text{gene}\;\text{in}\;\text{calibrator}\right)\;–\;\text{Ct}\left(\text{reference}\;\text{gene}\;\text{in}\;\text{calibrator}\right)$$

#### Histopathological, histochemical and morphometric examination

The midsection of the tibias were dissected out with sharp blade and fixed in 4% neutral buffered formaldehyde. Decalcification was performed in ethylene diaminetetra-acetic acid (EDTA) solution with PH 7 for about 4 weeks.

The EDTA was refreshed every 3 days until a fine needle could easily be inserted into the bone. Decalcified specimens were then washed, dehydrated in gradient alcohol, embedded in paraffin wax. The decalcified specimens were processed for paraffin blocks and serial transverse sections from the diaphysis were obtained.

Serial 5 μm thick sections were subjected to Haematoxylin & eosin (H&E) and Masson’s trichrome stain.

#### Statistical analysis

Data were coded and entered using the statistical package SPSS version 25. Data was summarized using mean and standard deviation. Comparisons between groups were done using analysis of variance (ANOVA) with multiple comparisons post hoc test. Correlations between quantitative variables were done using Pearson correlation coefficient. P-values less than 0.05 were considered as statistically significant.

## Results

### Comparison of the morphological findings which was detected in the tibias between the studied groups

**Fig 1:**
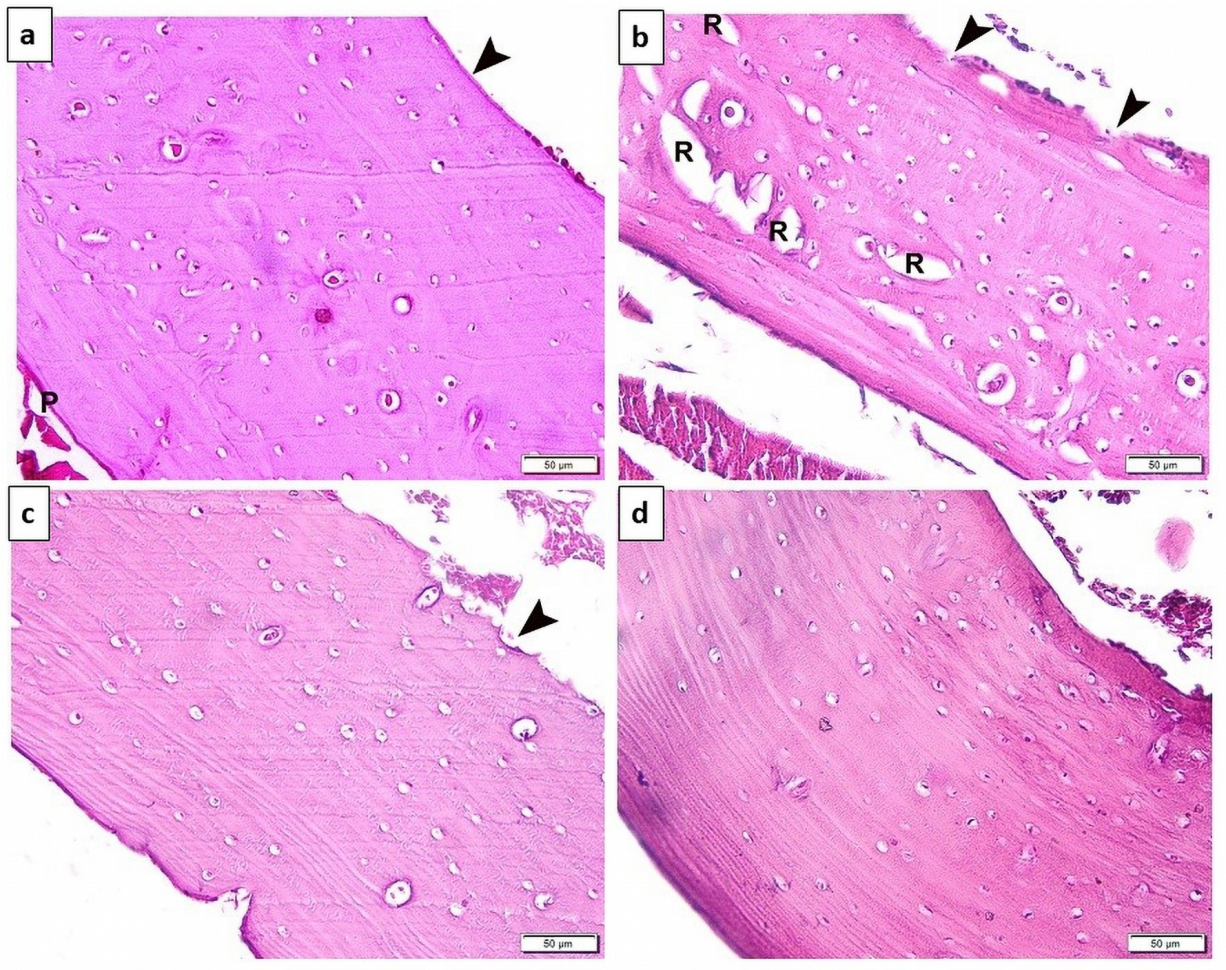
Fig (1-a) Photomicrograph of transverse section (TS) in the cortical compact bone of the tibia of the rat sham-operated control group, demonstrated normal structure of the cortical compact bone with outer periosteum (P), matrix containing blood vessels and osteocytes inside their lacunae and smooth endosteal surface (arrowhead). Fig (1-b): Photomicrograph of TS in the tibia of the untreated osteoporotic group showing: Multiple resorption cavities (R) and eroded endosteal surface. Fig (1-c) Photomicrograph of TS in the tibia of Carv-treated group showing apparently normal structure of the cortical bone with blood vessels and osteocytes inside their lacunae, yet eroded endosteal surface (arrowhead) was noted. Fig (1-d) Photomicrograph of TS in the tibia alendronate-treated showing preserved normal structure of the cortical compact bone. Scale bar 50 μm (H&E, x400).

Hematoxylin and Eosin examination (Fig 1) showed that the tibias of the untreated osteoporotic rats exhibited multiple resorption cavities and eroded endosteal surface (Fig 1b). The tibias of Carv-treated group showed apparently normal structure of the cortical bone with blood vessels and osteocytes inside their lacunae, yet eroded endosteal surface was noted (Fig 1c). Alendronate-treated rats had a preserved normal structure of their tibial cortical compact bone (Fig 1d).

**Fig 2:**
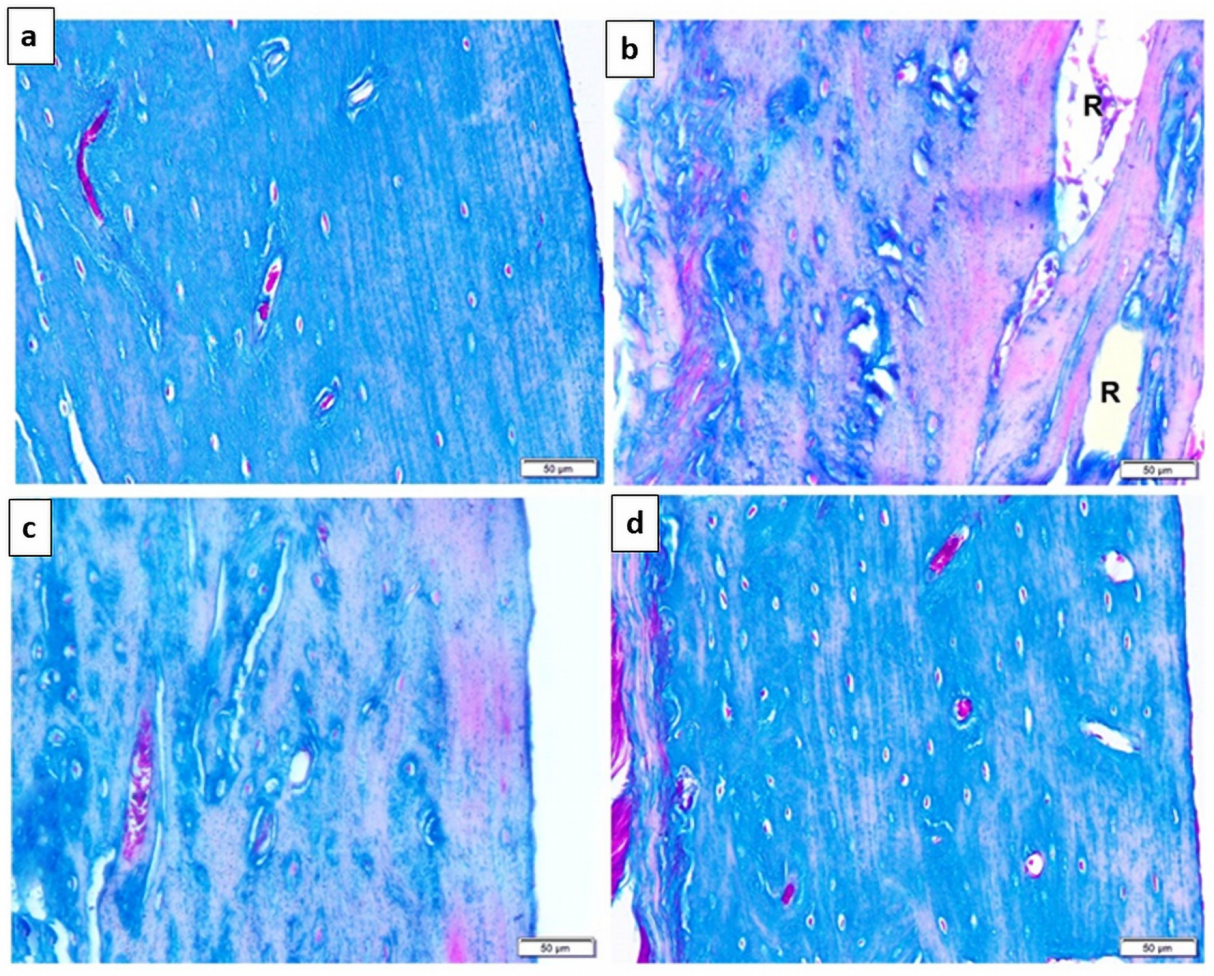
Photomicrograph of TS in the cortical compact bone of the tibia showing. (Fig 2-a) sham-operated control group: Regularly arranged bone matrix illustrating bluish-stained collagen. (Fig 2-b) untreated osteoporotic group: Resorption cavities (R) with obvious reduction of the bluish-stained collagen content in the bone matrix. (Fig 2-c) Carv-treated group: Increase in the collagen of the bone matrix. (Fig 2-d) Alendronate-treated group: Increase in the bluish-stained collagen of the bone matrix. Scale bar 50 μm. (Masson’s Trichrome, x200).

Masson’s Trichrome Stain examination (Fig 2) demonstrated that the bone tissue of the control rats (sham operated rats) (Fig 2a) displayed regularly arranged bone matrix mainly composed of collagen. The untreated osteoporotic group (Fig 2b) illustrated multiple cavities formation with substantial reduction of the collagen content in the bone matrix. However, Carv-treated group (Fig 2c) and alendronate-treated group (Fig 2d) showed increase in the collagen of the bone matrix.

### Bone turn over markers (Alkaline phosphatase, osteocalcin and pyridinoline)

Moreover, we observed a significant increase in the mean values of serum ALP, OC and pyridinoline in the untreated osteoporotic group as compared to their corresponding values in the control group, as shown in Table 2. We also observed a significant reduction in the same parameters in CARV treated group and alendronate treated group when compared with the untreated osteoporotic group, however, no significant change was noticed in the mean values of these parameters between the two treated groups and both drugs showed no significant difference in the level of pyridinoline as compared to the control group. Regarding serum osteocalcin, Alendronate-treated rats showed no significant difference in its level as compared to control rats while Carvedilol couldn’t bring it back to the control values.

**Table 2:** Comparison of the mean values of serum ALP, OC and pyridinoline among the studied groups.

	Control (n = 10)	Untreated osteoporosis group (n = 10)	CARV-treated osteoporosis group (n = 10)	Alendronate treated osteoporosis group (n = 10)
**ALP (IU/L)**	120±5.26	306.52±19.54 ^a^	176.24±9.07 ^a^ ^b^	157.88±4.13 ^a^ ^b^
**Osteocalcin (ng/ml)**	2.18±0.55	7.14±0.62 ^a^	3.85±0.5 ^a^ ^b^	3.27±0.75 ^b^
**Pyridinoline (nmol/L)**	1.78±0.5	6.99±1.89 ^a^	3.06±0.65 ^b^	3.11±0.77 ^b^

Table 2 showing a comparaison of the mean values of serum ALP, OC and pyridinoline among the studied groups Values are presented as mean ±SD;

^a^: Statistically significant compared to corresponding value in control group (P<0.05);

^b^: Statistically significant compared to corresponding value in Untreated osteoporosis group (P<0.05).

Comparisons between groups were done using analysis of variance (ANOVA) with multiple comparisons post hoc test.

### Protein expression of OPG, RANKL and gene expression of MMP-9 in tibia tissue among all the studied groups

The present results showed that induction of osteoporosis resulted in elevation of the mean values of tissue protein expression of RANKL and OPG and the gene expression of MMP-9 in untreated osteoporotic group when compared with sham control group as noticed in Table 3. These results may be denoting the potential role of these molecules in osteoporosis pathogenesis yet we found no significant changes in RANKL/OPG ratio among all groups.

**Table 3:** Comparison of the mean values of protein expression of OPG, RANKL and gene expression of MMP-9 among all the studied groups.

	Control (n = 10)	Untreated osteoporosis group (n = 10)	CARV-treated osteoporosis group (n = 10)	Alendronate treated osteoporosis group (n = 10)
**RANKL protein expression** (**pmol/mg)**	0.41±0.07	0.91±0.14 ^a^	0.51±0.04 ^b^	0.48±0.2 ^b^
**OPG protein expression** (**pmol/mg)**	10.54±1.22	19.66±1.57 ^a^	12.82±2.42 ^b^	12.96±1.59 ^b^
**RANKL/OPG ratio**	0.04±0.00	0.05±0.01	0.04± 0.01	0.04±0.02
**MMP_9 gene expression**	1.04±0.04	6.09±0.81 ^a^	2.95±0.66 ^a^ ^b^	2.5±1.18 ^b^

Table 3 showing a Comparison of the mean values of protein expression of OPG, RANKL and gene expression of MMP-9 among all the studied groups. Values are presented as mean ±SD;

^a^: Statistically significant compared to corresponding value in control sham-operated group (P<0.05);

^b^: Statistically significant compared to corresponding value in untreated osteoporosis group (P<0.05) Comparisons between groups were done using analysis of variance (ANOVA) with multiple comparisons post hoc test.

Our results also revealed in Table 3 a significant reduction in the mean values of protein expression of RANKL and OPG and the gene expression of MMP-9 in CARV treated osteoporosis group and alendronate treated group when compared with untreated osteoporotic group, with no significant variations in RANKL/OPG ratios among all groups. Both treated groups showed no significant variations in the mean values of these parameters as compared to each other and as compared to the sham operated group, except for MMP-9 gene expression that Carvedilol couldn’t bring it back to the control values.

### Correlations between MMP-9 gene expression and RANKL, OPG and bone turnover parameters among the studied groups

We noticed a significant positive correlation between MMP9 gene expression, RANKL, OPG protein expression, serum ALP, OC and pyridinoline (p<0.001, r = 0.792, p<0.001, r = 0.799, p<0.001, r = 0.906, p<0.001, r = 0.913 and p<0.001, r = 0.824 respectively).

### Lipid profile: (Serum cholesterol, TGs, HDL)

As shown in Table 4, the effect of ovariectomy operation on untreated osteoporotic group, yielded a highly significant increase in the mean values of serum cholesterol, TGs with a significant reduction in the protective HDL when compared with the corresponding values in control (sham-operated) group.

**Table 4:** Comparison of the mean values of serum cholesterol, TGs and HDL among all studied groups.

	Control (n = 10)	Untreated osteoporosis group (n = 10)	CARV-treated osteoporosis group (n = 10)	Alendronate treated osteoporosis group (n = 10)
**Cholesterol(mg/dl)**	132.4±7.54	203±9.14 ^a^	175.8±10.13 ^a^ ^b^	163.6±7.02 ^a^ ^b^
**TGs (mg/dl)**	64.8±8.79	103±6.32 ^a^	80±5.92 ^b^	76±10.56 ^b^
**HDL (mg/dl)**	54.88±4.79	33.38±3.47 ^a^	47.36±4.33 ^b^	48.1±4.56 ^b^

Table 4 showing a Comparison of the mean values of serum cholesterol, TGs and HDL among all studied groups. Values are presented as mean ±SD;

^a^: Statistically significant compared to corresponding value in control sham-operated group (P<0.05);

^b^: Statistically significant compared to corresponding value in untreated osteoporosis group (P<0.05) Comparisons between groups were done using analysis of variance (ANOVA) with multiple comparisons post hoc test.

The effect of CARV treatment in CARV-treated osteoporosis group yielded a significant decrease in the mean values of cholesterol and TGs and a significant elevation in the protective HDL. Alendronate treated group showed a significant decrease in the mean values of cholesterol and TGs with a significant increase in HDL level when compared with untreated osteoporotic group as shown in Table 4. However, no significant changes in the level of these markers were observed between the treated groups as compared to each other and as compared to the sham-operated control group, except that both drugs failed to return the serum cholesterol level back to the control values.

### Inflammatory indices: (NF-Қb, TNF, IL-6, CRP and Nitric oxide)

As regards the inflammatory indices, our results reported a marked significant elevation in the mean values of NF-Қb gene expression in tibial tissue, serum TNF-α, IL-6, hsCR and Nitric oxide as a result of ovariectomy and subsequent induction of osteoporosis in the untreated osteoporosis group when compared with corresponding values in control sham-operated group as shown in Table 5 denoting the dramatic effect of osteoporosis on inflammatory indices.

**Table 5:** Comparison of the mean values of serum TNF-α, IL-6, hsCR and Nitric oxide among all the studied groups.

	Control (n = 10)	Untreated osteoporosis group (n = 10)	CARV-treated osteoporosis group (n = 10)	Alendronate treated osteoporosis group (n = 10)
**NF_KB gene expression**	1.04±0.02	5.12±0.77 ^a^	2.04±0.22 ^a^ ^b^	2.01±0.39 ^a^ ^b^
**TNF-α (ng/ml)**	17.34±3.07	107.54±8.11 ^a^	50.26±17.21 ^a^ ^b^	43.14±20.27 ^b^
**IL_6 (ng/ml)**	35.48±5.45	114.7±10.35 ^a^	77.02±10.68 ^a^ ^b^	63.62±11.71 ^a^ ^b^
**CRP(ng/ml)**	0.99±0.06	4.32±1.39 ^a^	1.94±0.64 ^b^	1.99±0.56 ^b^
**NO (μmol/l**)	15.06±2.79	71.42±16.48 ^a^	31.32±8.88 ^b^	31.86±5.23 ^b^

Table 5 showing a comparison of the mean values of serum TNF-α, IL-6, hsCR and Nitric oxide among all the studied groups. Values are presented as mean ±SD;

^a^: Statistically significant compared to corresponding value in control sham-operated group (P<0.05);

^b^: Statistically significant compared to corresponding value in untreated osteoporosis group (P<0.05) Comparisons between groups were done using analysis of variance (ANOVA) with multiple comparisons post hoc test.

We also noticed a remarkable significant reduction in the mean values of all these parameters in CARV treated group and in alendronate treated group when compared with untreated osteoporotic group, denoting the anti-inflammatory effect of both drugs. There was no significant difference in these parameters between the treated groups as compared to each other. Both drugs couldn’t return NF-Қb gene expression and IL-6 to the control values, while the levels of serum CRP and NO showed no significant difference among the control and both treated groups. Regarding TNF-α, while alendronate-treated group showed no significant difference in its value as compared to control sham-operated group, Carvedilol failed to bring its value back to the control values as shown in Table 5.

### Correlations between MMP-9 gene expression and parameters of lipid profile, inflammation, NF-Kb parameters among the studied groups

To further emphasize the relation of MMP-9 gene expression with other parameters, correlation studies were done. On reviewing the results of correlation to identify factors most strongly correlated with MMP9 expression, beside the significant correlations previously illustrated between MMP-9 gene expression and bone turnover markers and mediators (tissue protein expression of RANKL, OPG, serum ALP, OC and pyridinoline), the present study revealed also a significant correlation between MMP-9 gene expression and dyslipidemia and inflammation markers (serum levels of TC, TGs, HDL, serum TNF-α, IL6, CRP, nitric oxide, gene expression of NF-KB), which represent major risk factors for CVD.

A significant positive correlation was noticed between MMP9 gene expression and serum TC and TGs as a result of osteoporosis induction (p <0.001, r = 0.877 and p <0.001, r = 0.835 respectively) while HDL showed a negative correlation with MMP_9 gene expression (p <0.001, r = -0.830).

And, a significant positive correlation was also noticed between MMP9 gene expression and gene expression of NF-KB, serum levels of TNF-α, IL6, CRP and nitric oxide (p<0.001, r = 0.876, p<0.001, r = 0.952, p<0.001, r = 0.852, p<0.001, r = 0.821 and p<0.001, r = 0.886 respectively).

Taken together, these results provide a solid evidence about the pivotal involvement of MMP-9 in the pathophysiology of both OP and CVD.

## Discussion

Ovariectomized (OVX) rat represents an excellent preclinical animal model that properly emulates the important clinical features of estrogen depleted human skeleton and the response to therapeutic agents [29].

The present study was conducted on a post-menopausal osteoporosis rat model, which was confirmed in the untreated OVX group by histological examination of tibial bones. Hematoxylin and eosin (H&E) staining demonstrated that bilateral OVX resulted in osteoporosis with loss of bone mass of tibias, eroded endosteal surface, remarkably decreased trabeculae with a loosely arranged structure were observed in the metaphysis, remarkable increase of the marrow fat (adipogenesis) in addition to marked bone resorption represented by presence of numerous osteoclasts on the surface of the trabeculae. Moreover masson tichrome stain of tibias of the untreated OVX group showed obvious reduction of collagen content in the bone matrix denoting bone resorption. Furthermore, analysis of bone turnover markers in the untreated OVX group showed a significant increase serum levels of ALP, OC (bone forming markers) and pyridinoline (bone resorping marker) as compared to the control sham operated group.

The currently accepted mechanism of skeletal bone loss with estrogen deficiency is the imbalance in bone turnover, where bone resorption exceeds bone formation [30]. The proposed mechanisms for bone loss include RANKL upregulation which leads to increased osteoclast recruitment and activation; and decreased osteoclast apoptosis. On the other hand, the elevated OPG production by osteoblasts aims to compensate and neutralize the RANKL bone resorbing effect, but, finally RANKL production exceeds the OPG compensating effect and favours bone resorption and subsequent osteoporosis [31].

Indeed, the present work showed a significant elevation in tibial protein expression of RANKL and OPG levels in untreated OVX group as compared to SHAM operated group. This is in accordance with Kai Luo et al., [32]. In another study, Li et al., [33] reported an elevated RANKL mRNA expression but a reduced OPG mRNA expression level in the tibias when compared with SHAM group.

The correlation between OPG/RANKL levels and the risk of osteoporotic fractures is still controversial [34]. Low serum OPG was found in vertebral fractures in postmenopausal osteoporotic women [35] while another study noticed increased risk of hip and wrist fractures in women with high serum OPG [36]. A prospective population analysis showed that low serum RANKL levels were associated with a high risk of atraumatic fracture regardless of age, sex, menopausal status, OPG level, and lifestyle [37]. Yet, in Chinese Women Aged 20–75, Liu et al. found that neither serum levels of OPG nor RANKL or RANKL/OPG ratio correlated with BMDs after adjustment of age and menopause, and they showed no differences among normal, osteopenic and osteoporotic postmenopausal women [38].

Intriguingly, in our study, the RANKL/OPG ratio showed no significant difference among groups, yet MMP 9 gene expression revealed a significant elevation, in tibias of untreated osteoporotic rats as compared to the control sham-operated group, and, a significant positive correlation was elucidated between MMP9 and OPG, RANKL expression among the studied groups.

These results support a previous study by Zheng et al., [39] who found that MMP-9 mRNA expression level in the OVX group increased abruptly.

Accumulated evidence indicated that MMPs serve a critical role in osteoclastic bone resorption and facilitate the migration of osteoclasts to bone surfaces via the extracellular matrix [40]. MMP-9, is essential for initiating the osteoclastic resorption process in cases of osteoporosis by removing the collagenous layer from the bone surface before demineralization can start [41]. Recently, MMP-9 has been also reported to be necessary for the regulation of gene pathways that are required for osteoclastogenesis, through its proteolytic interaction with the histone H3 N-terminal tail [42].

As regards the association between MMP9 expression and bone turnover markers, our results noticed a positive correlation between MMP9 and ALP, osteocalcin and pyridinoline in agreement with previous studies [43,44].

Taken together, as compared to RANKL/OPG, MMP-9 could be considered a simpler and more sensitive marker for osteoporosis and potentially for increased cardiovascular diseases risk as will be discussed further.

Dyslipidaemia is a major risk factor for the development of cardiovascular diseases [45].

Analysis of the lipid profile in our untreated OVX rats showed a significant elevation in serum levels of total cholesterol (TC), triglycerides (TGs), and a significant reduction in protective high density lipoprotein (HDL) as compared to sham-operated rats. These results are in agreement with Jianfeng Han and Wei Wang, [46]. On the contrary, Solomon et al., [47] found no association between BMD and serum lipid concentrations, and Brownbillet al., [48] found even a positive relationship between BMD and dyslipidaemia markers.

While the explanation of these contradictory results are yet to be explored, our study support the main body of evidence that suggest a positive link between low BMD and dyslipidaemia, especially in postmenopausal women [49].

Interestingly, our results exhibited a significant positive correlation between MMP-9 expression in tibias and serum levels of TC, TGs and a significant negative correlation between its expression and serum levels of HDL. Far to our knowledge, we are the first to study the correlation between serum dyslipidaemia and MMP-9 gene expression in bones as a probable molecular connection between osteoporosis and cardiovascular diseases risk factors.

Results of the present work revealed also that, the serum concentrations of inflammatory markers hsCRP, TNF-α and IL-6 in the untreated OVX rats were increased as compared to sham-operated rats. These results are in corroboration with the study of Orsal and his colleagues [50] on ovariectomized rats and Pasco et al, [50] study on elderly women. Indeed, both osteoporosis and atherosclerosis have been linked to an overall inflammatory state [51] and the levels of CRP, IL-6, and TNF-α directly correlated to the bone resorptive action of monocytes [52].

Interestingly, our results noticed a significant positive correlation between MMP9 tissue expression and inflammatory markers in all studied groups which agree with Zhang et al., [53] who noticed a positive association between MMP9 and TNF-α in osteoporosis in humans, and Singh et al., [54] who showed NF_Kb as an upstream event in CRP-mediated MMP-9 induction in rat models of inflammatory diseases.

Alendronate, a nitrogen-containing bisphosphonate (BPs), is considered the most potent drug that inhibits bone resorption and became the treatment of choice for osteoporosis [37]. Emerging evidence has suggested that alendronate may have potential cardiac protecting effects [55], and recently, it is also linked with reduced risk of cardiovascular death, heart attack, and stroke [56]. Accumulating experimental studies have suggested the ability of BPs to reduce the formation of atherosclerotic plaques and to inhibit vascular calcifications, but the results of these studies are contrasting and the exact mechanism is not fully clear [57].

Our work revealed also a protective role of alendronate on dyslipidaemia and inflammatory markers in alendronate treated OVX rats when compared with the untreated osteoporotic rats.

Adami et al., [58] was the first to report a significant reduction in TC and an increase in high-density lipoprotein-cholesterol (HDL-C) in postmenopausal women treated with alendronate. Guney et al., [59] also revealed the reducing effect of bisphosphonates on total cholesterol, triglyceride and LDL-cholesterol levels but not HDL-cholesterol in osteoporotic hyperlipidaemia females. In contrast, these positive effects on lipids have not yet been confirmed by the majority of studies carried out with N-BPs [60].

As regards inflammatory indices, in agreement with our results, many studies have shown that the cells most sensitive to bisphosphonates are those of the monocyte/macrophage system, in which osteoclasts are included. It has been reported that bisphosphonates inhibit monocyte/macrophage migration, suppress antigen presentation by cells of the monocyte/macrophage lineage, and inhibit the production of a variety of bone-resorbing cytokines, including TNFα [61]. Moreover, Corrado et al., [62] reported the CRP-reducing effect of alendronate in various experimental animal models and in human studies.

Interestingly, our work revealed that alendronate treatment significantly reduced tissue level of MMP9 gene expression in ovariectomized rats when compared with their counterpart untreated group. Melani et al., [63] has also detected a Bisphosphonate–Mediated MMP-9 Inhibition while studying BALB-neuT mice.

To conclude, Alendronate reduced significantly MMP-9, improved significantly CVD risk factors and efficiently treated osteoporosis as evident by histological examination and BTM levels in Alendronate-treated group as compared to the untreated osteoporotic rats and the control sham-operated rats. MMP-9 has been significantly correlated with atherosclerosis risk factors (dyslipidaemia and inflammatory markers) as well as with bone turnover markers in this study on osteoporosis rat model, as it has been previously demonstrated by our group while studying an atherosclerosis rat model [10], which supports our hypothesis that suggests MMP-9 as a key molecule that links these two pathologies.

Thus, one of the important aims of the present study was to examine the possible protective effect of Carvedilol, one of the medications for atherosclerosis, on osteoporosis and to evaluate the effects of this drug on MMP-9 activity.

Bones are innervated by sympathetic neurons that promotes bone resorption and inhibit osteoblast proliferation [64]. Although CARV is not superior to traditional β-blockers in blood pressure control, it still shows great benefit in the inhibition of collagen deposition [65,66]. Therefore, Carvedilol may protect against osteoporosis.

Interestingly, the CARV-treated group showed an apparently normal histopathological structure of the cortical bone with blood vessels and osteocytes inside their lacunae, yet an eroded endosteal surface was noted by H&E stain. In addition, Masson’s trichrome stain noticed an increase in collagen content of the bone matrix denoting the possible protective effect of this drug in attenuating the osteoporotic findings.

Moreover, a significant reduction of tibial protein expression of RANKL and OPG levels was detected in CARV treated osteoporotic rats as compared with the untreated OVX rats. This is in accordance with Da Liu [67] and with Arau´ jo et al., [68] who noticed a significant reduction in RANKL expression in rat model of periodontitis treated with CARV, but the latter group found an elevation in OPG levels.

Importantly, our results showed a significant reduction in bone turn-over markers (serum levels of ALP, OC and pyridinoline) in CARV treated osteoporotic group when compared to the untreated group in accordance with Boshra and El Wakeel [13], and a significant reduction in serum levels of CRP, TNF alpha, IL-6, NO and NF-KB in CARV treated OVX rats as compared to the untreated osteoporotic rats in agreement with previous studies [68,69].

Taken together our results illustrated CARV beneficial effect not only on attenuating bone loss but also on reducing biomechanical fragility and bone resorbing cytokines.

Our results showed also a significant decrease in MMP9 expression in tibial bone of CARV-treated osteoporotic rats compared to their counterpart untreated group in agreement with previous studies in different animal models [10,68].

To our knowledge, the present study is the first to evaluate the effect of CARV treatment on MMP9 gene expression in osteoporosis induced rat model.

Interestingly, in our study, CARV effect on bone was significant yet partial, as evident by H&E and the serum levels of BTM as the markers in CARV treated rats didn’t reach the control values although significantly improved as compared to the untreated osteoporotic rats. In fact, this matches the effect of CARV treatment on MMP-9, as it also results in significant yet partial improvement in MMP-9 expression.

So, taken together, and considering the high significant correlation between MMP-9 and both BTM & CVD risk factors among all studied groups, our results support our hypothesis that propose MMP-9 as a molecular link between both pathologies and shows the promising potential effect of targeting MMP-9 on bone and CVD simultaneously. More epidemiological and analytical studies are needed to validate these assumptions.

## Conclusions

Pathogenesis of CV events and OP are strikingly overlapping. Although RANKL/OPG axis has been suggested to be the potential pathophysiological link for both atherosclerosis and bone loss, yet because of the complexity of the system, the literature raise controversial results yet to be clarified. This study presents MMP-9, a downstream molecule executing the destructive aspect of RANKL/OPG system, as a simple molecular link more consistently associated with the pathophysiology of both osteoporosis and CVD risk factors. Based on this hypothesis, a holistic approach to treatment is encouraged to involve the use of drugs that may have beneficial effects on bone as well as on CVD, probably through targeting MMP-9, which was further investigated in this study, where alendronate attenuated CVS risk factors namely dyslipidaemia and inflammation and where carvedilol also exerted a bone preservative role in accordance with their respective effect on MMP-9.
